# Thrombocytopenia with Absent Radii (TAR) Syndrome Without Significant Thrombocytopenia

**DOI:** 10.7759/cureus.10557

**Published:** 2020-09-20

**Authors:** Jael Cowan, Taral Parikh, Rajdeepsingh Waghela, Ricardo Mora

**Affiliations:** 1 Pediatrics, Woodhull Medical Center, Brooklyn, USA; 2 Neonatology, Woodhull Medical Center, Brooklyn, USA

**Keywords:** thrombocytopenia with absent radii (tar) syndrome, hypo-megakaryocytic thrombocytopenia, bilateral absent radii

## Abstract

Thrombocytopenia with absent radii (TAR) syndrome is a rare genetic syndrome that occurs with a frequency of about 0.42 cases per 100,000 live births. It is characterized by hypo-megakaryocytic thrombocytopenia with bilateral absent radii and the presence of both thumbs. The thrombocytopenia is initially very severe, manifesting in the first few weeks to months of life, but subsequently improves with time to reach near normal values by one to two years of age. We present a case of a newborn with TAR syndrome with an atypical presentation of mild thrombocytopenia in the first week of life, with early normalization of platelet counts in the neonatal period. The patient deviates from the normal pattern in which 95% of patients with TAR syndrome usually develop significant thrombocytopenia (platelet counts of less than 50 x 10 ^9 ^platelets/L) within the first four months of life. Additionally, the absence of hypo-megakaryocytes on peripheral smear sets this patient apart from the typical cases of TAR syndrome.

TAR syndrome is often associated with significant morbidity and mortality secondary to severe thrombocytopenia, which occurs with the highest frequency in the first 14 months of life. The most common cause of mortality is due to a severe hemorrhagic event occurring in the brain, gastrointestinal tract, and other organs. Therefore, all patients with TAR syndrome should be monitored closely for symptomatic thrombocytopenia with platelet transfusions being implemented as the first-line therapy for the treatment of severe or symptomatic disease.

## Introduction

Thrombocytopenia with absent radii (TAR) syndrome is a rare congenital defect characterized by thrombocytopenia with specific skeletal abnormalities, primarily of the upper limbs. It was first characterized as a syndrome by Hall et al. in 1969, who delineated the diagnostic criteria for TAR syndrome as the association of thrombocytopenia with bilateral absent radii and the presence of both thumbs [1]. The presence of both thumbs is a characteristic feature that distinguishes TAR syndrome from other conditions associated with similar skeletal abnormalities such as Fanconi anemia and Holt-Oram syndrome.

The overall prevalence of TAR syndrome is estimated to be 1:200,000-1:100,000 [2].

TAR syndrome rarely occurs in the United States, so its frequency in the United States is unknown. Additionally, TAR syndrome is usually characterized by the development of significant thrombocytopenia within the first few weeks of life, usually with platelet counts less than 50 x 10 ^9^ platelets/L [3].

We report a rare case of a newborn with a diagnosis of TAR syndrome without significant thrombocytopenia.

## Case presentation

A full-term, appropriate-for-gestational-age female neonate was admitted to the neonatal intermediate care unit at the Woodhull Hospital for observation due to prenatal concerns for a possible diagnosis of TAR syndrome. The neonate was born to a 34-year-old G6P3023 female who received early and adequate antenatal care at a prenatal clinic at the Wyckoff Hospital. The mother took prenatal vitamins throughout the course of her pregnancy. She was fully immunized and denied a history of drug/substance use during her pregnancy. Routine maternal serology was unremarkable, including a Quad screen done at 17 weeks of gestation. However, a prenatal sonogram performed at 22 weeks of gestation, and an amniocentesis at 26 weeks of gestation had findings suggestive of TAR syndrome. The exact findings were not readily available since the prenatal care was done at an outside facility.

The pregnancy course was significant for a urinary tract infection (UTI) in the second trimester, which was successfully treated with nitrofurantoin. Additionally, the mother developed preterm contractions at 33 weeks of gestation and was hospitalized for two days. She received antenatal steroids for fetal lung maturation, magnesium sulfate for neuroprotection, and antibiotic coverage due to her group B streptococcus (GBS) unknown status. She was subsequently discharged and re-admitted at 38 weeks of gestation in active labor.

The neonate was delivered via elective cesarean section due to concerns of a possible low platelet count in the neonate secondary to TAR syndrome. APGARs (Appearance, Pulse, Grimace, Activity, and Respiration) were 8/9 at 1 and 5 minutes, respectively. The neonate received routine resuscitation at birth, and had a birth weight of 3015 g, length 48 cm, and head circumference of 33.5 cm.

**Figure 1:**
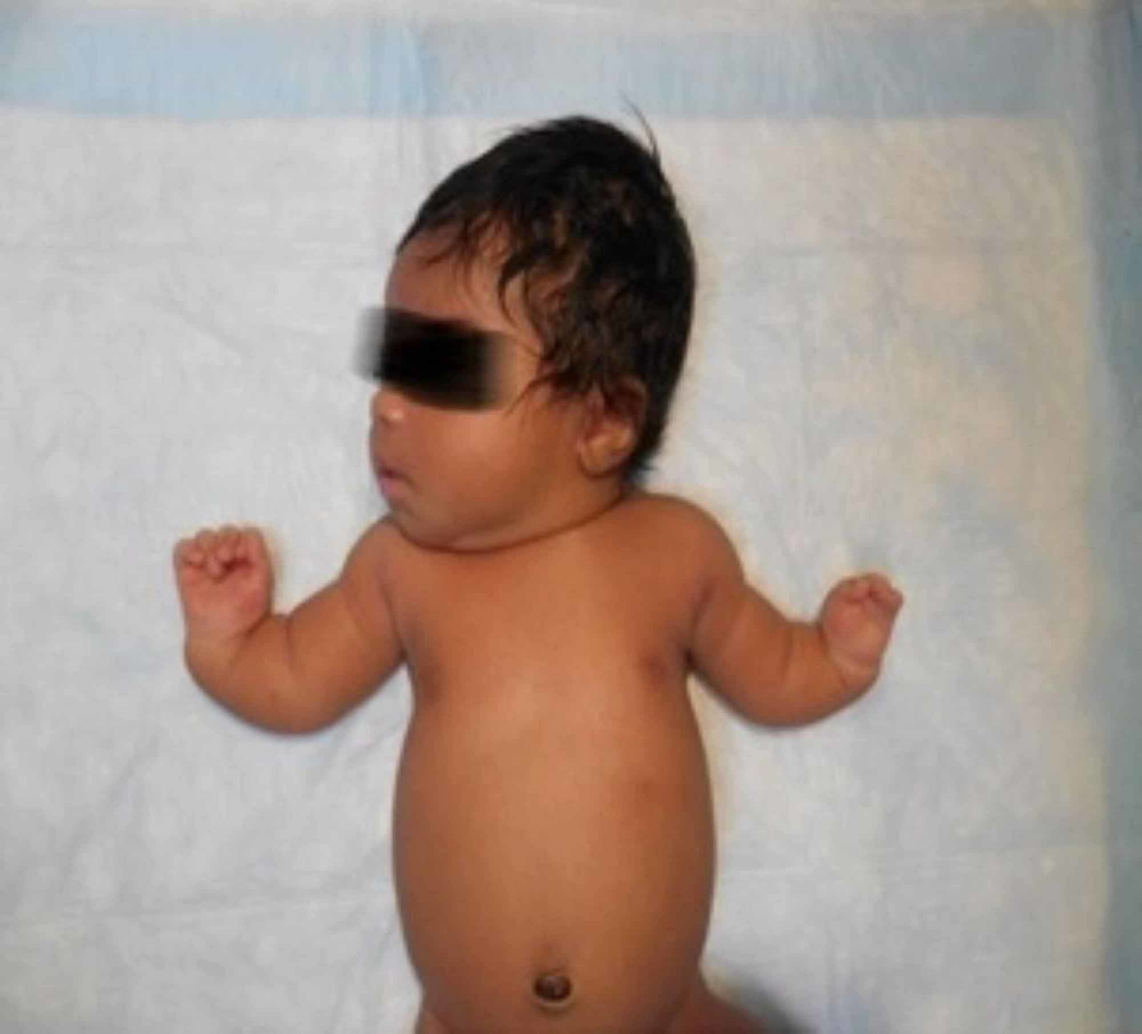
Shortening of the forearms, varus angulation of hands towards midline with presence of the thumbs bilaterally

The physical examination was significant for upper extremity abnormalities, including bilateral shortening of the forearms, bilateral varus angulation of hands towards midline with the presence of the thumbs bilaterally (Figure 1).

There was also syndactyl of the digits 1-4 of the left hand and a web between the thumb and 4th digit. No abnormality of the lower extremities was noted. The ears were noted to be low set bilaterally, but the infant had no other dysmorphic features. Examination of all other systems were within normal limits.

Postnatal evaluation included serial platelet monitoring, peripheral smear, X-rays of the upper extremities, abdominal ultrasound, echocardiogram, and genetic studies.

**Figure 2:**
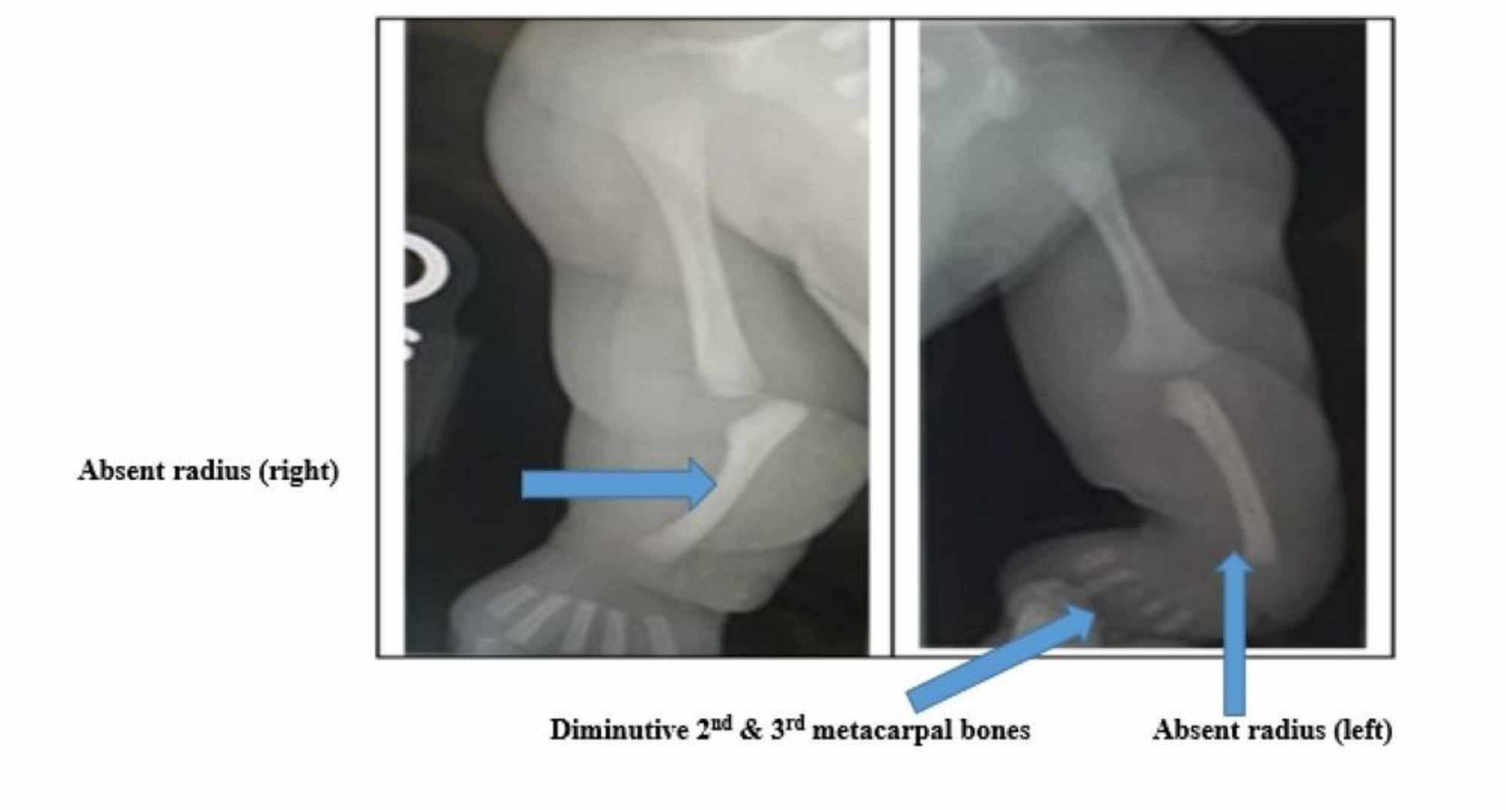
X-ray of upper extremities demonstrating bilateral absence of the radii

X-rays of the upper limbs demonstrated bilateral absence of the radii with varus angulation of the metacarpal bones relative to the ulna and diminutive second and third metacarpal bones of the left hand (Figure 2).

**Figure 3:**
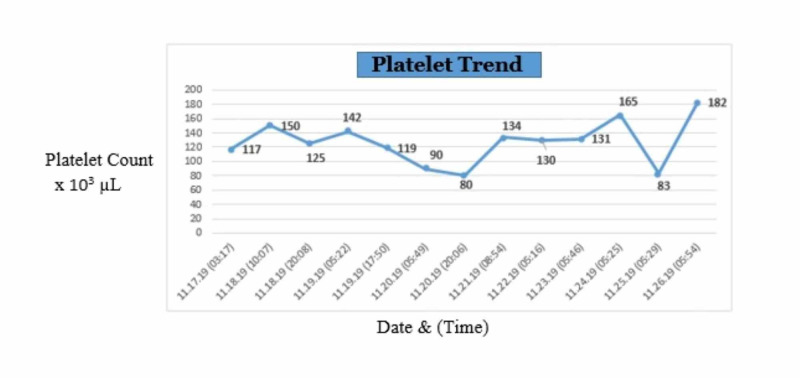
Platelet trend

Serial complete blood count (CBC) was done to monitor platelet counts which ranged from 80,000 - 182,000 platelets/µL over the patient’s-day hospital course (Figure 3).

**Figure 4:**
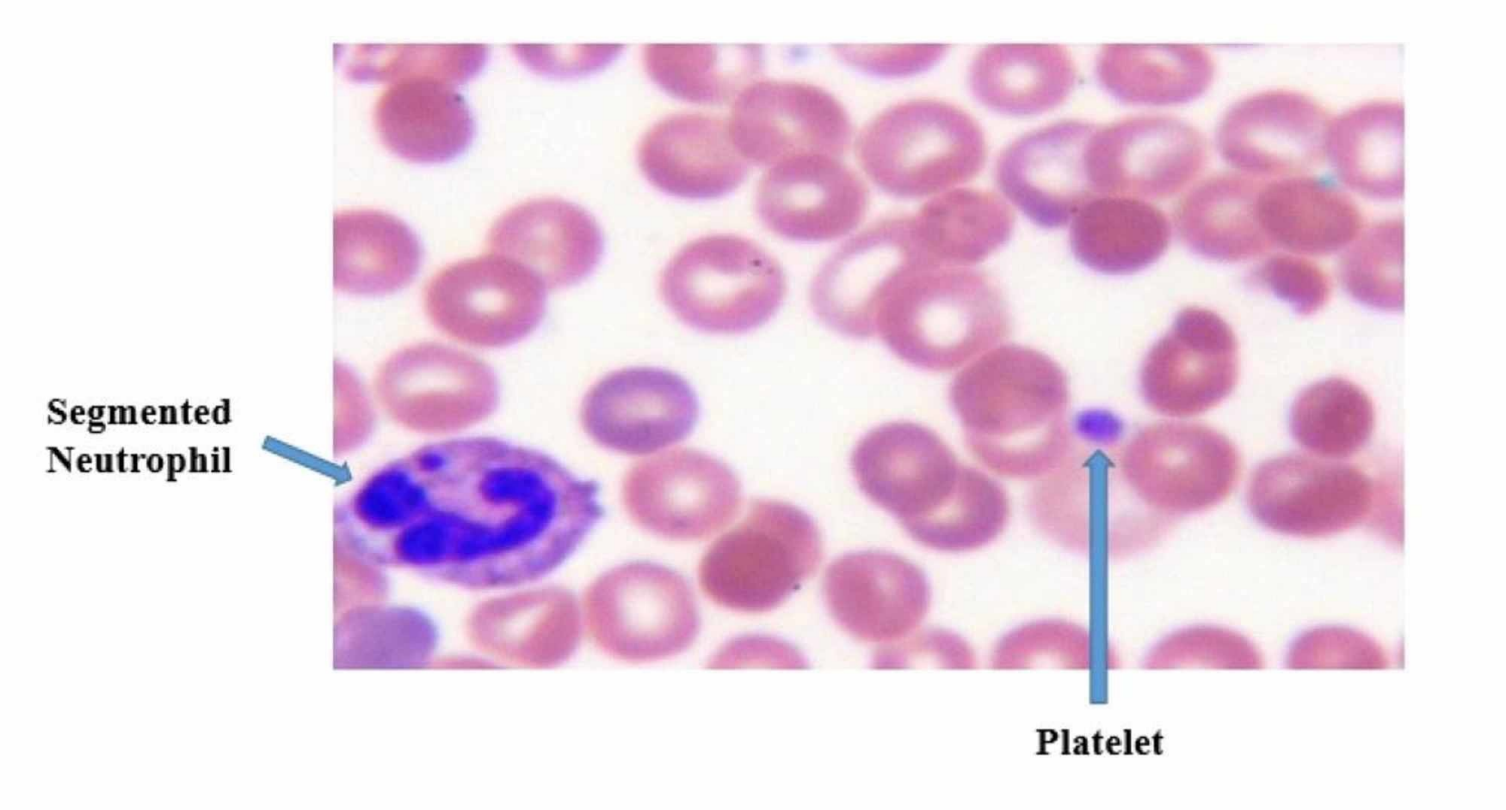
Peripheral smear

The peripheral smear showed normal platelet morphology without the presence of hypo-megakaryocytes (per Hematology review) (Figure 4).

Imaging studies included an abdominal ultrasound and an echocardiogram. The abdominal ultrasound was done to evaluate the kidneys which appeared normal in size and echotexture, without evidence of hydronephrosis. The echocardiogram showed a small patent foramen ovale (PFO) with left to right shunt, but was otherwise normal.

Genetic testing was performed with findings of a normal female karyotype (46 XX) on chromosomal analysis and a pathogenic deletion of 2.4 Mb detected on chromosome 1q21.1 with Online Mendelian Inheritance in Man (OMIM) genes RNA-binding motif protein 8A (RBM8A), peroxisomal biogenesis factor 11 beta (PEX11B), gap junction protein alpha 5 (GJA5), gap junction protein alpha 8 (GJA8) included in the deletion on molecular cytogenetic analysis.

The infant’s hospital course and management was guided by a multi-disciplinary team comprised of Neonatologists, Pediatric Cardiologist, Pediatric Hematologist, as well as social workers. The neonate was started on expressed breast milk and formula soon after birth. A hypoallergenic formula was given, per Pediatric Hematology recommendation, due to the association between TAR syndrome and cow milk protein allergy. Serial CBC done to monitor the patient’s platelet count demonstrated only mild thrombocytopenia with a nadir of 80,000 platelets/µL on day three of life. No evidence of bleeding or other clinical manifestations of thrombocytopenia was noted.

**Figure 5:**
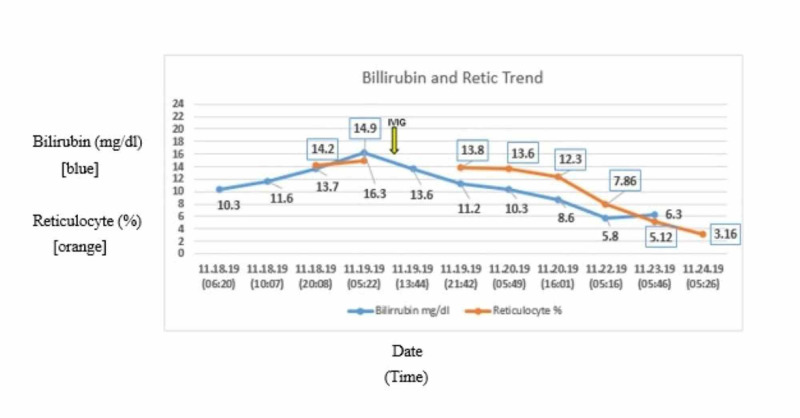
Bilirubin and reticulocyte trend

However, the infant’s clinical course was significant for hyperbilirubinemia requiring phototherapy treatment, which was noted on day 2 of life. The hyperbilirubinemia was accompanied by a high reticulocyte count (max 14.9%) in the absence of any major blood group incompatibility. The patient received one dose of intravenous immunoglobulins (IVIG) with improvement in both the hyperbilirubinemia and the reticulocyte count (Figure 5).

The neonate was eventually discharged on day 9 of life, with appointments for follow up with Pediatric Hematology, Genetics, and Orthopedics.

At present, the patient continues to be followed up in the outpatient clinic with ongoing monitoring of the platelet count. The patient’s platelet count normalized on day nine of life, and has remained greater than 150,000 platelets/µL at follow up visits; as such the Hematologist has recommended for CBC checks to be done on a three-monthly schedule. Additionally, the patient was reviewed by Orthopedics who recommended reconstructive surgery at one year of life.

## Discussion

TAR syndrome is a rare congenital defect characterized by hypo-megakaryocytic thrombocytopenia and bilateral radii aplasia or hypoplasia in the presence of both thumbs. The presence of both thumbs distinguishes TAR syndrome from other conditions associated with phocomelia, such as Fanconi anemia and Holt-Oram syndrome.

The etiology of TAR syndrome is unknown, however, a study by Klopocki et al. demonstrated that all individuals born with TAR syndrome possessed a microdeletion of the 200 kilo-base (kb) region at the chromosome band 1q21.1 [4]. This microdeletion in the long (q) arm of chromosome 1 usually involves deletion of a gene called the RBM8A gene. However, this microdeletion alone is not enough to cause the onset of TAR syndrome. TAR syndrome usually occurs when the chromosome 1q21.1 microdeletion is associated with another genetic anomaly. The presence of an RBM8A hypomorphic allele in conjunction with the 1q21.1 microdeletion was found to be significantly associated with TAR syndrome.

Co-inheritance of the 1q21.1 microdeletion (which includes the RBM8A gene of that chromosome), together with a single nucleotide polymorphism (SNP) in a specific region of the other RBM8A gene is thought to be the underlying molecular defect in TAR syndrome [5]. This results in a reduction in the total amount of RNA-binding motif protein 8A, a protein encoded by the RBM8A gene. The RNA-binding motif protein 8A is theorized to be instrumental in facilitating various cellular functions and producing proteins related to fetal tissue development. The lack of these proteins is associated with TAR syndrome; however, the specific mechanism as to why this occurs is unknown [2,6].

The pathophysiology of the thrombocytopenia in TAR syndrome is due to impaired maturation of megakaryocyte progenitor cells in the bone marrow, resulting in hypo-megakaryocytic thrombocytopenia. Megakaryopoiesis is the process by which bone marrow progenitor cells differentiate into mature megakaryocytes, which eventually produce platelets. Several studies have attempted to explain the pathophysiology underlying the impaired megakaryopoiesis seen in TAR syndrome, however to date this is still unclear. Several biological and molecular studies in TAR syndrome have demonstrated increased levels of the cytokine Thrombopoietin (TPO) with suboptimal (in vitro) differentiation of megakaryocyte progenitor cells in response to TPO, suggesting a possible defect in the TPO signaling pathway [3]. TPO and its receptor c-mpl are required for the commitment of megakaryocyte progenitor cells towards differentiated megakaryocytes. In a study by Ballmaier et al., TPO levels were found to be elevated in patients with TAR syndrome compared to healthy subjects, suggesting a lack of response to the action of TPO in the signal transduction pathway of megakaryopoiesis and thrombocytopoiesis. However, the expression of the TPO receptors (c-mpl) was found to be normal, similar to that of controls [7]. These findings raised questions regarding a possible defect in the c-Mpl signaling pathway; however, further studies to understand the genetic basis of TAR syndrome have failed to identify mutations in the c-mpl gene in affected patients indicating that the problem was elsewhere. In other studies, the RBM8A gene mutation has been shown to be associated with impaired megakaryopoiesis. Albers et al. discovered a low-frequency SNP in the noncoding 5’ untranslated region of the RBM8A gene. This mutation causes a significant reduction in the level of a protein called Y14, which is encoded by the RBM8A gene [6]. Other studies have shown an SNP in the first intron of the RBM8A gene produces similar effects [5]. This Y14 protein is a key component of the exon junction splicing complex; however, the exact mechanism by which a reduction in Y14 causes the hypo-megakaryocytic thrombocytopenia of TAR syndrome remains unclear.

The inheritance pattern of TAR syndrome is uncertain. Previous studies have suggested an autosomal recessive pattern of inheritance due to findings of multiple children in the same family, born to the same parents, being diagnosed with TAR syndrome [4]. However, more recent studies suggest that the inheritance of TAR syndrome may actually follow an autosomal dominant pattern with variable penetrance. In an epidemiological study of TAR syndrome conducted in Spain by Martinez-Frias et al., the incidence of TAR syndrome was found to be 0.42 cases per 100,000 live births. The sex ratio of diagnosed infants was 1:1 [8].

Individuals with TAR syndrome are found to have phocomelia of both upper limbs, although it may also involve the lower limbs. The thumbs of affected individuals are found to be held in flexion against the palm and have a concomitant limited functionality [9]. The majority of infants with TAR syndrome develop thrombocytopenia within the first week of life, and 95% do so within the first four months of life with platelet counts usually less than 50 x 10 ^9^ platelets/L [3]. The thrombocytopenia is initially very severe (less than 30 x 10 ^9^ platelets/L), but the platelet count slowly improves with time to reach near normal values by one to two years of age [5]. The degree of thrombocytopenia is proportional to the severity of symptoms associated with it, and the development of a petechial rash and bleeding typically signifies a falling thrombocyte count. The mortality rate in TAR syndrome is dependent on the patient’s age and platelet count. The most common cause of mortality in patients with TAR syndrome is a severe hemorrhagic event occurring in the brain, gastrointestinal tract, and other organs. One study by Hedberg et al. revealed that most deaths occurring secondary to a hemorrhagic events occurred with a platelet count of less than 10 x 10 ^9^ platelets/L. However, the incidence of hemorrhage is generally limited to the first 14 months of life [10].

TAR syndrome is a clinical diagnosis based upon the findings of a bilateral absent radii with the presence of both thumbs, along with thrombocytopenia of generally less than 50 x 10 ^9^ platelets/L. Additionally, neonates suspected to have TAR syndrome are often diagnosed in the intrapartum period of the pregnancy during routine fetal scans, in which hypoplastic or aplastic radii are observed. Diagnosis can be confirmed via molecular genetic testing to detect the microdeletion in the 1q21.1 chromosomal band. Quantitative polymerase chain reaction (PCR) and gene-targeted microarray designed to detect single-exon deletions or amplifications may both be used to investigate the gene defects. In addition, gene-targeted studies can be performed to reveal a deletion or duplication of the RBM8A gene [2,6].

The diagnosis of TAR syndrome in our index case was made presumptively during the intrapartum period, then confirmed postnatally by clinical examination and genetic testing. Our patient had the classic physical manifestations of TAR syndrome. However, the anticipated drop in platelet count to levels of less than 50 x 10 ^9 ^platelets/L within the first few weeks of life did not occur in our patient. Additionally, our patient had early normalization of platelet count within the neonatal period, and the peripheral smear did not demonstrate the hypo-megakaryocytic picture that is typical of TAR syndrome; likely explaining the absence of significant thrombocytopenia noted in this case.

The initial management of patients suspected to have TAR syndrome involves serial postnatal monitoring of platelet counts. Platelet transfusions are given as needed for thrombocytopenia [11]. Bone marrow transplant is not indicated, given the course of the thrombocytopenia is fluctuant, and the platelet count generally stabilizes after two years of life. Platelet stimulating agents such as romiplostim and oprelvekin have shown promising results in their ability to correct thrombocytopenia in patients affected by TAR syndrome [12]. Orthopedic intervention with procedures such as prosthesis, orthoses, and adaptive device is indicated to manage the absent radii [13]. In addition, it is recommended that cow’s milk should be avoided in patients with TAR syndrome, as most suffer from an associated cow’s milk protein allergy. To prevent allo-immunization secondary to serial platelet transfusions, it is recommended that after two years of life, platelet infusions should only be given if the platelet count falls below 10 x 10 ^9 ^platelets/L [2].

TAR syndrome has also been found to be associated with cardiac abnormalities such as tetralogy of Fallot and atrial septal defects [14], genitourinary tract anomalies such as renal dysgenesis, and rarely, Mayer-Rokitansky-Kuster-Hauser syndrome [15].

## Conclusions

TAR syndrome is often a clinical diagnosis based upon the findings of bilateral absent radii with the presence of both thumbs, along with thrombocytopenia of generally < 50 x 10 ^9^ platelets/L. The diagnosis can be confirmed by genetic testing demonstrating a microdeletion on chromosome 1q21.1, with or without SNP involving a low-frequency noncoding region of the RBM8A gene. The thrombocytopenia seen in TAR syndrome is a hypo-megakaryocytic thrombocytopenia that may be present at birth, or may develop transiently in the first few weeks to months of life. It is generally fluctuant in nature, but typically ameliorates by two years of life. However, this case report describes a unique case of TAR syndrome without significant thrombocytopenia (< 50 x 10 ^9^ platelets/L) occurring in the first few months of life, and early normalization of the platelet count in the neonatal period.

However, due to the significant morbidity and mortality associated with severe thrombocytopenia, all patients with TAR syndrome should be monitored closely for symptomatic thrombocytopenia with the intervention of platelet transfusions as needed for severe/symptomatic disease. Additionally, these patients should be screened for co-existing congenital anomalies in the heart and genitourinary tract, with appropriate referrals as needed. Treatment typically requires a multi-disciplinary team approach to ensure adequate and successful management of patients with TAR syndrome.
